# The genome sequence of *Dermanura phaeotis *(Chiroptera, Phyllostomidae, Stenodermatinae; Miller, 1902)

**DOI:** 10.12688/wellcomeopenres.27248.1

**Published:** 2026-09-02

**Authors:** Melissa R de Waal, Nancy B Simmons, Brian P O'Toole, Ning Zhang, Jonathan L Gray, Erick Duarte, Linelle Abueg, Samuel C Talbot, Kirsty McCaffrey, Bonhwang Koo, Giulio Formenti, Meike Mai, Myrtani Pieri, Erich D Jarvis, Emma C Teeling, Sonja C. Vernes, The Bat1K Consortium

**Affiliations:** 1 Department of Biological Sciences, Fairleigh Dickinson University, Madison NJ 07940, USA; 2 Division of Mammals,, Department of Vertebrate Zoology, National Museum of Natural History, Smithsonian Institution, Washington, DC 20560, USA; 3 Department of Mammalogy, Division of Vertebrate Zoology, American Museum of Natural History, New York NY 10024, USA; 4 Paratus Sciences, New York NY, USA; 5 Vertebrate Genome Lab, The Rockefeller University, New York NY, USA; 6 School of Biology, University of St Andrews, St Andrews, UK; 7 Department of Life Sciences, School of Life and Health Sciences, University of Nicosia, 2417, Nicosia, Cyprus; 8 School of Biology and Environmental Science, University College Dublin, Dublin, Ireland; 9 Wellcome Genome Campus, Wellcome Sanger Institute, Cambridgeshire, England, UK

**Keywords:** Dermanura phaeotis, genome sequence, chromosomal, Bat1K

## Abstract

We present a genome assembly from an individual male *Dermanura phaeotis* (Chordata; Mammalia; Chiroptera; Phyllostomidae). The genome sequence is 2.2 Gb in span. The majority of the assembly is scaffolded into 30 chromosomal pseudomolecules, with the X and Y sex chromosomes assembled.

## Species taxonomy

Eukaryota; Metazoa; Chordata; Craniata; Vertebrata; Euteleostomi; Mammalia; Eutheria; Laurasiatheria; Chiroptera; Yangochiroptera; Phyllostomidae; Stenodermatinae; Stenodermatini; Artibeina; *Dermanura*; *Dermanura phaeotis* ( Baker et al., 2016; Cirranello et al., 2016; Simmons and Cirranello, 2025).

## Introduction

*Dermanura* is a clade of small, stripe-faced frugivorous bats in the Subfamily Stenodermatinae. For many years these bats were treated as a subgenus of *Artibeus* (e.g., Koopman, 1994; Simmons, 2005) but more recently have been treated as distinct at the genus level ( Mammal Diversity Database, 2026; Simmons and Cirranello, 2025). *Dermanura* bats are common throughout the Neotropics with multiple species often occurring in sympatry ( Emmons and Feer, 1997; Reid, 2009). As shown in Figure 1, *Dermanura phaeotis* currently includes 3 subspecies, *D. p. phaeotis* (Atlantic side of Central America from Veracruz [Mexico] to Costa Rica), *D. p. nanus* (western Mexico from Sinaloa to Guerroro), and *D. p. palatinus* (Pacific side of Central America from Oaxaca, Mexico, to Costa Rica) ( Koopman, 1994; Simmons and Cirranello, 2025). It does not include *Dermanura rava (= ravus)*, which was previously treated as a subspecies of *phaeotis* (e.g., Koopman, 1994; Simmons, 2005) but which is now regarded as a distinct species ( Hoofer et al., 2008; Solari et al., 2009; Mammal Diversity Database, 2024; Simmons and Cirranello, 2025). As currently recognized, *Dermanura phaeotis* occurs in Mexico, Belize, Guatemala, Honduras, El Salvador, Nicaragua, Costa Rica, and possibly in Panama and Colombia ( Koopman, 1994; Mammal Diversity Database, 2024). Reports of this species from elsewhere in northern South America may be in error and may date from the time when *rava* was included in *phaeotis.*

**Figure 1.**
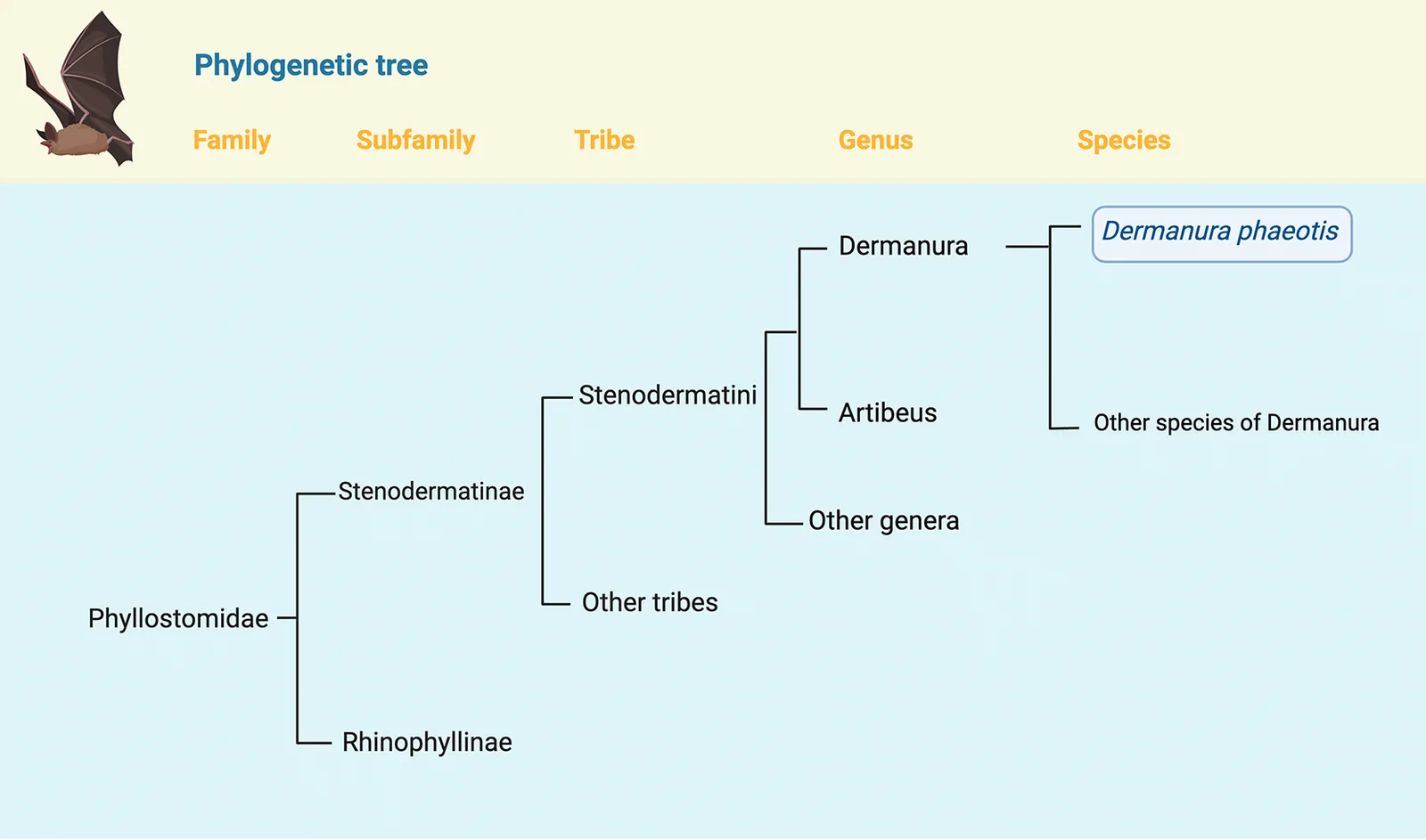
Position of *Dermanura phaeotis* in the phylogeny of Family Phyllostomidae. *Dermanura phaeotis* is one of 11 species currently recognized in the genus *Dermanura* ( Simmons and Cirranello, 2025). *Dermanura* belongs to the Tribe Stenodermatini in the Subfamily Stenodermatinae, which currently includes 19 genera and 76 species ( Baker et al., 2016; Cirranello et al., 2016; Simmons and Cirranello, 2025). Phylogeny based on Hoofer et al. (2008), Solari et al. (2009), and Rojas et al. (2016).

Like other members of the genus *Dermanura, D. phaeotis* ( Figure 2) has brown fur and black wing membranes, white facial stripes, a broad muzzle, a horseshoe-shaped upper tooth row, and central upper incisors that are bilobed and less than twice as long as the outer incisors ( Emmons and Feer, 1997; Reid, 2009). *Dermanura phaeotis* can be distinguished from congeners based on a series of morphological traits including the following: small size (forearm 35–40 mm; greatest length of skull 17–21 mm; 9–15 g); dorsal fur faintly tricolored and sandy brown or gray-brown; fur moderately short and smooth (4–6 mm on upper back; prominent white facial stripes that are crisply outlined with upper stripe extending to the front of the ear; ear pinnae and noseleaf pale brown and edged with yellow or white; horseshoe of noseleaf free from upper lip; uropatagium U-shaped (9–11 mm) with dorsal surface almost naked; uropatagium not fringed though may have a few short hairs; postorbital and preorbital processes absent; last upper and lower molars absent; dental formula 2/2, 1/1, 2/2, 2/2 x 2 = 28 ( Timm, 1985; Koopman, 1994; Reid, 2009).

**Figure 2.**
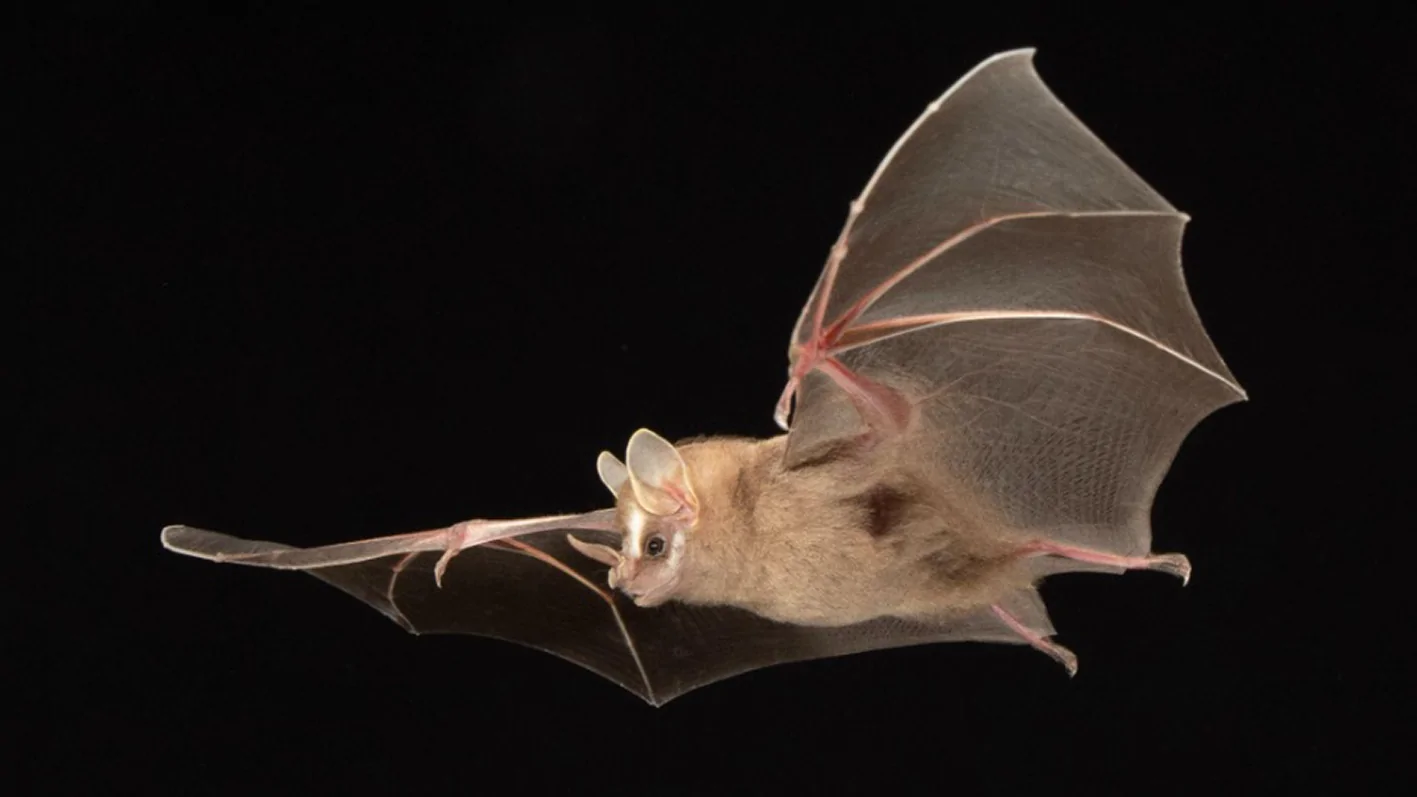
Dermanura phaeotis. *Dermanura phaeotis* in flight showing coloration, facial stripes, and noseleaf. [Photos taken at Lamanai, Belize by Charles Francis].

*Dermanura phaeotis* is locally common and is found in lowland evergreen and deciduous forests up to 1,200 m, and it also occurs in secondary forest and disturbed habitats ( Timm, 1985; Reid, 2009; Miller et al., 2015). *Dermanura phaeotis* feeds on fruits from understory trees and shrubs but also occasionally eats pollen and insects ( Reid, 2009; Miller et al., 2015; Ingala et al., 2021). It is a tent-making bat that roosts under modified palm, banana, and *Heliconia* leaves ( Timm, 1985, 1987; Reid, 2009; Miller et al., 2015; García-Méndez and Santos-Moreno, 2020; López-González, 2020).

*Dermanura phaeotis* is classified as Least Concern in the IUCN Redlist of Threatened Species because of its wide distribution, presumed large population size, occurrence in a number of protected areas, tolerance to some degree of habitat modification, and because it is unlikely to be declining at the rate required to qualify for listing in a threatened category ( Miller et al., 2015).

## Genome sequence report

The genome was sequenced from a single male *Dermanura phaeotis* (field number BZ-12, catalog number AMNH:Mammalogy:280606 collected at the Lamanai Outpost Lodge, Indian Church, Orange Walk District, Belize, on 8 November 2021. A total of 25-fold coverage in Pacific Biosciences Hi-Fi long reads (contig N50 57.9 Mb) was generated after removal of all reads shorter than 10 kb. Primary assembly contigs were scaffolded with chromosome confirmation Hi-C data. The final assembly has a total length of 2.2 Gb in 106 sequence scaffolds with a scaffold N50 of 154 Mbp ( Table 1 **).** The majority, 99.4%, of the assembly sequence was assigned to 30 chromosomal-level scaffolds, representing 14 autosomes (numbered by sequence length, and the X + Y sex chromosomes). Chromosomal pseudomolecules in the genome assembly of *Dermanura phaeotis* are shown in Table 2. The assembly has a BUSCO ( Simão et al., 2015) completeness of **97.8**% using the vertebrata odb10 reference set. The assembly is fully phased, and both haplotypes are deposited therein.

**Table 1.** Genome data for *Dermanura phaeotis.*

*Project accession data*	
Assembly identifier	mDerPha1.hap1
Species	*Dermanura phaeotis*
Specimen	muscle, liver, heart, testes, spleen, kidney
NCBI taxonomy ID	40229
BioProject	Bat1K: Accession: PRJNA489245; ID: 489245
BioSample ID	SAMN48779402
Isolate information	male
***Raw data accessions***	
Pacific Biosciences SEQUEL II	SRR34021296, SRR34021294, SRR34021292, SRR34021290 (.bam) SRR34021295, SRR34021293, SRR34021291, SRR34021289 (.fastq)
Hi-C Illumina	SRR34021298, SRR34021297
Assembly accession	GCA_051107565.1
Accession of Alternative haplotype	GCA_051107595.1
Span (Gb)	2.2
Number of contigs	182
Contig N50 length (Mb)	58
Number of scaffolds	105
Scaffold N50 length (Mb)	154.6
Longest scaffold (Mb)	246.0

* BUSCO scores based on the mammalia_odb10 BUSCO set using v5.0.0. C = complete [S = single copy, D = duplicated], F = fragmented, M = missing, n = number of orthologues in comparison.

* ***Dermanura phaeotis*** BUSCO scores based on laurasiatheria_odb10 BUSCO set v5.3.2.

**Table 2.** Chromosomal pseudomolecules in the genome assembly of *Dermanura phaeotis.* ENA accession Chromosome Size (Mb) GC%. The chromosome number of *Dermanura phaeotis* is 2n = *30.*

ENA accession	Chromosome	Size (Mb)	GC%
CM117851.1	1	245.98	40.5
CM117852.1	2	214.80	40
CM117853.1	3	192.65	42
CM117854.1	4	179.79	43
CM117855.1	5	176.00	41.5
CM117856.1	6	154.62	43.5
CM117857.1	7	151.63	41.5
CM117865.1	X	143.49	41
CM117858.1	8	140.91	41
CM117859.1	9	127.78	43.5
CM117860.1	10	118.37	45
CM117861.1	11	110.21	42.5
CM117862.1	12	98.05	40
CM117863.1	13	57.58	46.5
CM117864.1	14	55.41	45.5
CM117866.1	Y	44.57	45.5

## Methods

The *Dermanura phaeotis* specimen was a male individual collected on an American Museum of Natural History (AMNH) field expedition near Lamanai Archaeological Reserve in the Orange Walk District of Belize. The individual sampled was identified as *D. phaeotis* based on morphological traits (e.g., noseleaf structure and color, fur color and stiping) as described above. The bat was caught in a ground-level mist net set in the gardens of Lamanai Outpost Lodge (17.75156 N, 88.65376 W). All efforts were made to minimize any distress or suffering by the animal. The individual sample was subjected to minimal handling after capture, and it was held in a clean cloth bag after capture as per best practices for field containment of bats. After species identification, the individual was euthanized humanely the same night it was captured. The animal was euthanized by isoflurane inhalation (Formula CHF _2_OCClHCF _3_, CAS number 26675–46-7; Manufacturer Piramal Critical Care, Supplier US Pharmacy Systems, Product code 5034-1FL-SOL-ORA), an approved and humane euthanasia method that rapidly causes unconsciousness and eventually death. Bats euthanized by this method are rendered unconscious within seconds due to their high respiration rate, and death occurs within a minute or two with no significant suffering by the animal. Capture and sampling were conducted under Belize Forest Department Permit FD/WL/1/21(16) and samples were exported under Belize Forest Department permit FD/WL/7/22(07). All work was conducted with approval by the AMNH Institutional Animal Care and Use Committee (AMNHIACUC-20210614). All data were recorded and reported in accordance with the ARRIVE 2.0 guidelines ( Percie du Sert et al., 2020) – see data availability section and Table 1.

Tissues were removed from the subject individual immediately following euthanasia and were flash-frozen in a liquid nitrogen dry shipper, with the cold chain maintained from field to museum to laboratory.

DNA was extracted using Nanobind extraction from **liver** tissue following the Circulomics Nanobind HMW DNA Extraction Protocol. Pacific Biosciences HiFi libraries were constructed according to the manufacturer’s instructions. Hi-C data was generated using the Arima Hi-C+ High Coverage kit from the same tissue sample. Sequencing was performed by the Genomic Operations DNA Pipelines at Paratus Sciences on Pacific Biosciences Sequel IIe (HiFi reads) and Illumina NextSeq 2000 (Hi-C) instruments.

Assembly was carried out following the Vertebrate Genome Project pipeline v2.0 ( Larivière et al., 2024) with a few modifications as follows. An initial QC analysis was performed on the raw BAM file using FastQC ( https://www.bioinformatics.babraham.ac.uk/projects/fastqc/). BAM files were converted to fastq format and merged for downstream processing. Genome size was estimated using GenomeScope2 ( Vurture et al., 2017). Hifiasm was used for genome assembly ( Cheng et al., 2021). The quality of the assembly was evaluated using Merqury ( Rhie et al., 2020) and BUSCO ( Manni et al., 2021). Scaffolding with Hi-C data ( Rao et al. 2014) was carried out with YaHs ( Zhou *et al.* 2023). PretextView was implemented to generate Hi-C contact maps. Figure 3 **–** Figure 6 were generated using BlobToolKit ( Challis et al. 2020). Software utilised for the *Dermanura phaeotis* analysis are depicted in Table 3.

**Figure 3.**
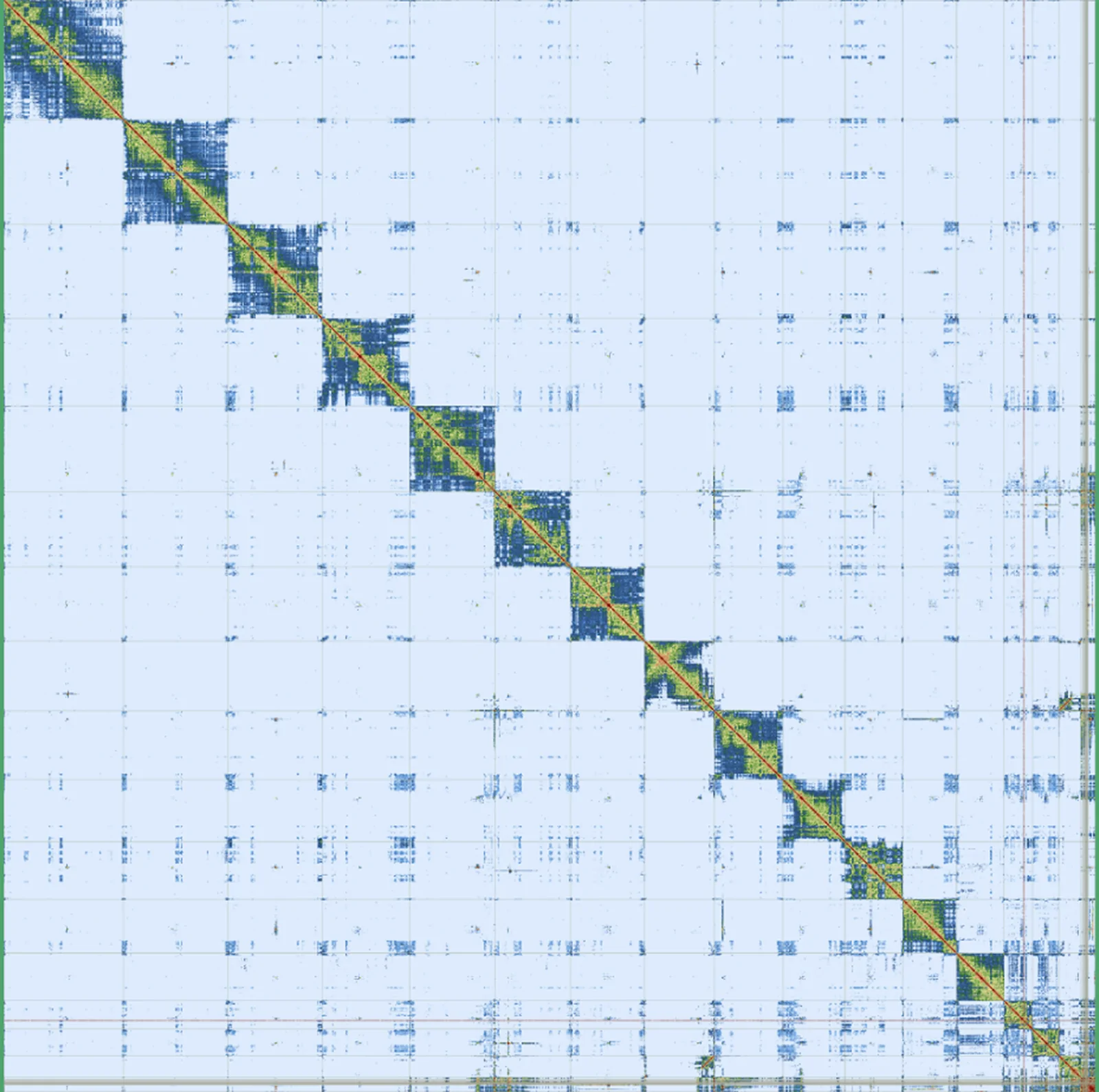
Hi-C Contact Map of the *Dermanura phaeotis* assembly with 16 chromosomes, visualized using PretextView.

**Figure 4.**
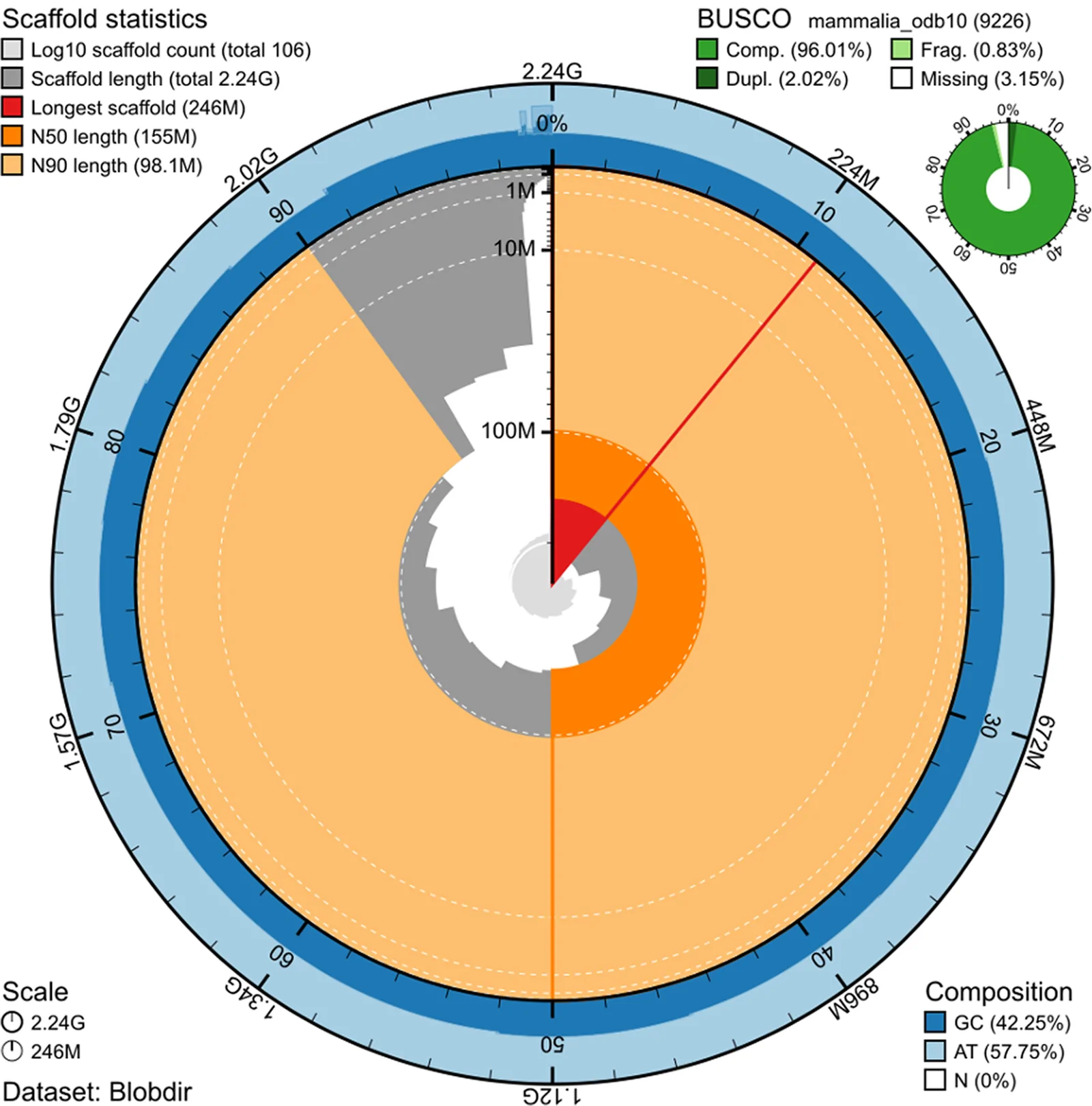
Genome assembly metrics generated using blobtoolkit for the *Dermanura phaeotis* genome assembly. The larger snail plot depicts scaffold statistics including N50 length (bright orange) and base composition (blue). The smaller plot shows BUSCO completeness in green.

**Figure 5.**
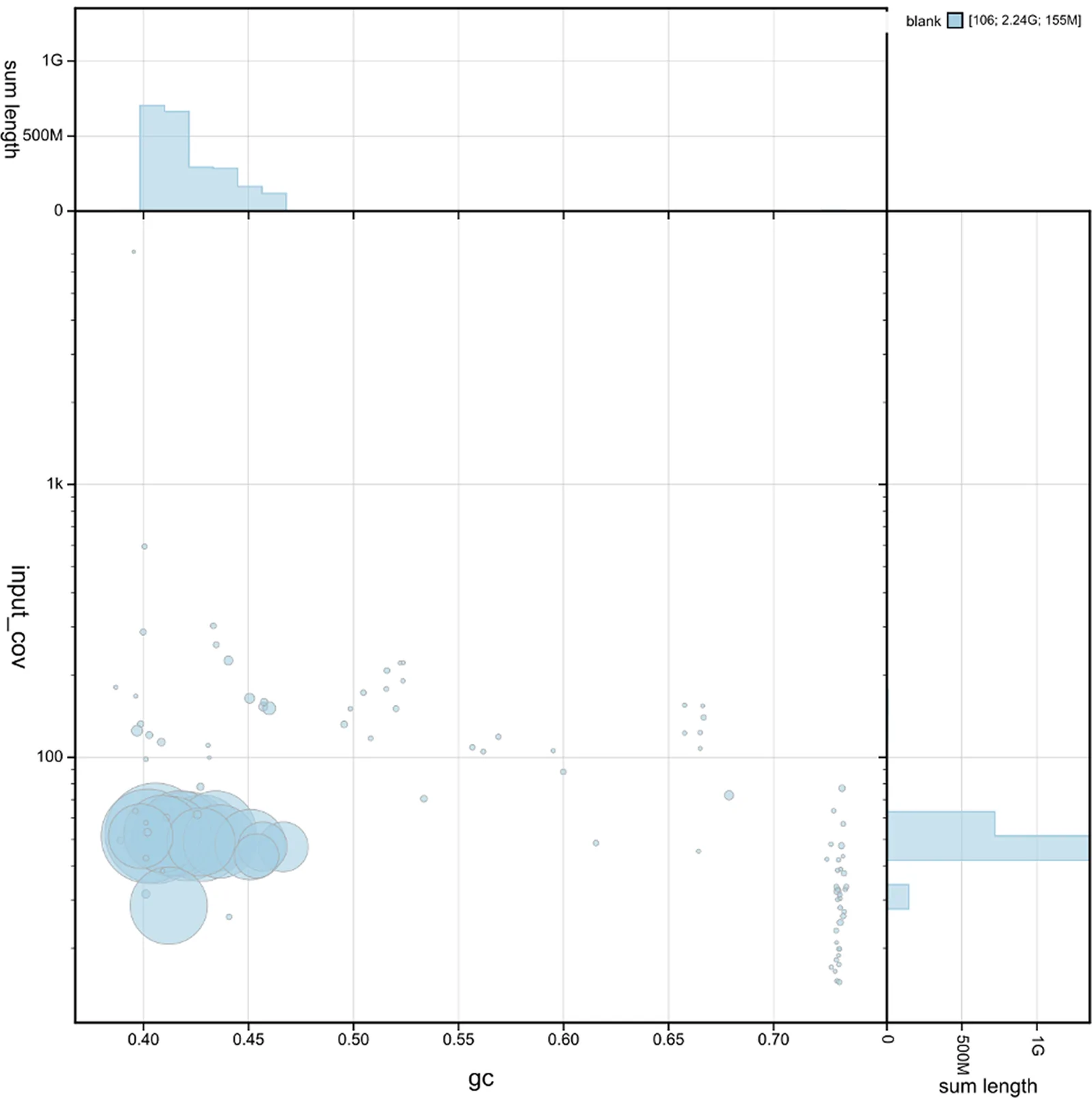
GC coverage plot generated for the *Dermanura phaeotis* assembly using blobtoolkit. Individual chromosomes and scaffolds are represented by each circle. The circles are sized in proportion to chromosome/scaffold length. Histograms show the sum length of chromosome/scaffold size along each axis. Color of circles indicate taxonomic hits of each Phylum represented in the assembly.

**Table 3.** Software tools used.

Software tool	Version	Source
bamUtil	1.0.15	https://genome.sph.umich.edu/wiki/BamUtil:_bam2FastQ
FastQC	0.11.9	https://www.bioinformatics.babraham.ac.uk/projects/fastqc/
MultiQC	1.11	https://github.com/ewels/MultiQC
Genomescope	2.0	https://github.com/tbenavi1/genomescope2.0
Hifiasm	0.19.3	https://github.com/chhylp123/hifiasm
BUSCO	5.3.2	https://busco.ezlab.org/
Merqury	1.3	https://github.com/marbl/merqury
gfastats	1.3.6	https://github.com/vgl-hub/gfastats
Arima-HiC Mapping Pipeline	-	https://github.com/ArimaGenomics/mapping_pipeline
YaHS	1.2a	https://github.com/c-zhou/yahs
samtools	1.9	https://www.htslib.org/
BlobToolKit	4.5.3	https://github.com/blobtoolkit/blobtoolkit
PretextView	1.0.0	https://github.com/sanger-tol/PretextView/releases/tag/1.0.0
BLAST	2.10.1	https://ftp.ncbi.nlm.nih.gov/blast/executables/blast+/2.10.1/
Kraken2	2.1.1	https://github.com/DerrickWood/kraken2
BWA-MEM2	2.2.1	https://github.com/bwa-mem2/bwa-mem2

**Figure 6.**
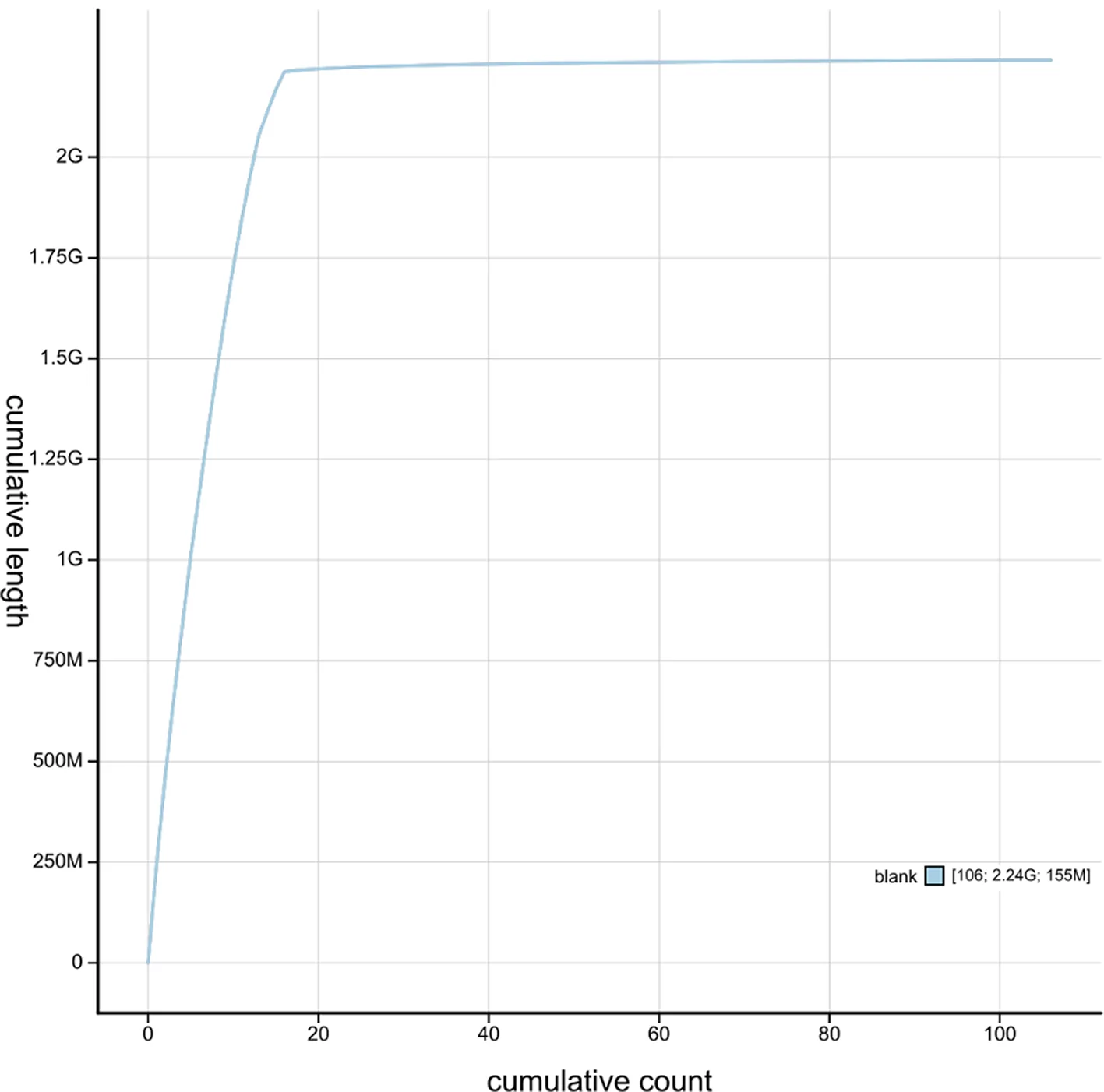
Cumulative sequence plot generated for the *Dermanura phaeotis* assembly using blobtoolkit. The grey line shows the cumulative length for all chromosomes/scaffolds in the assembly. Colored lines represent Phylum represented in the assembly.

### Ethics

The specimen used for genome sequencing was collected in the Orange Walk District of Belize under Belize Forest Department Permit FD/WL/1/21(16). Samples were exported under Belize Forest Department Permit FD/WL/7/22(07). All animal procedures were conducted with approval from the American Museum of Natural History Institutional Animal Care and Use Committee (AMNHIACUC-20210614).

The individual was captured using a ground-level mist net and handled in accordance with established best practices for the safe capture and handling of wild bats. Following species identification, the animal was euthanized humanely using isoflurane inhalation overdose. All efforts were made to minimise animal suffering and distress. The study is reported in accordance with ARRIVE 2.0 guidelines.

## Data Availability

The *Dermanura phaeotis* genome sequencing initiative is part of the Bat1K genome sequencing project. The genome assembly is released openly for reuse. The genome assembly can be found in the European Nucleotide Archive: Dermanura phaeotis. Accession number: GCA_051107565.1, https://www.ebi.ac.uk/ena/browser/view/GCA_051107565.1 NCBI BioProject: *Dermanura phaeotis* isolate: mDerPha1. Accession number: PRJNA1276126, https://www.ncbi.nlm.nih.gov/bioproject/PRJNA1276126/ Data accession identifiers are reported in Table 1. Zenodo. *The genome sequence of Dermanura phaeotis* (Chiroptera, Phyllostomidae, Stenodermatinae; Miller, 1902). DOI: https://doi.org/10.5281/zenodo.21207700. (Bat1K Consortium, 2026). This project contains the following extended data:
• ARRIVE 2.0 Checklist. Data are available under the terms of the Creative Commons Attribution 4.0 International Licence (CC BY 4.0).
