# Supplementary material for: The genome sequence of Dermanura phaeotis (Chiroptera, Phyllostomidae, Stenodermatinae; Miller, 1902)
Source: Wellcome Open Res. 2026 Sep 2;11:571. [Version 1] doi: 10.12688/wellcomeopenres.27248.1 (PMC13627936; doi:10.12688/wellcomeopenres.27248.1)
Supplement: Peer review history

# Reviewer report: Reviewer response for version 1

**DOI:** 10.21956/wellcomeopenres.29990.r170813

**Published:** 2026-10-12

**Authors:** Armin Scheben

**Affiliations:** 1 The Jackson Laboratory - NYSCF, New York, New York, USA

The authors present a brief report on the genome assembly of *Dermanura phaeotis *using the standard VGP bioinformatics pipeline. This is a useful resource for the community with no major issues. I have listed several minor points the authors may want to address below.

 - In the Methods, the authors indicate that "Assembly was carried out following the Vertebrate Genome Project pipeline v2.0 (Larivière et al., 2024) with a few modifications as follows." However, it is unclear which modifications these were because most of the steps listed in the following text seem consistent with the VGP 2.0 pipeline (Hifiasm for assembly, Hi-C scaffolding with YaHs, Merqury for quality assessment). I suggest the authors revise the passage to make it clearer which modifications were actually made and which steps are just brief descriptions of the VGP pipeline. Otherwise, the tools and version numbers used are well documented and cited.

 - The authors state "As shown in Figure 1, *Dermanura phaeotis* currently includes 3 subspecies,". However, Figure 1 does not show that D. phaeotis includes any subspecies. Please modify the figure to show this or revise the text.

 - As another reviewer pointed out, conformation is misspelt in "Primary assembly contigs were scaffolded with chromosome confirmation".

 - Some words in the manuscript may have been accidentally written in bold font ("97.8%" and "liver").

 - The Figure 5 y-axis label could be written out to clarify what exactly "input_cov" is.

Are sufficient details of methods and materials provided to allow replication by others?

Yes

Is the rationale for creating the dataset(s) clearly described?

Yes

Are the datasets clearly presented in a useable and accessible format?

Yes

Are the protocols appropriate and is the work technically sound?

Yes

Reviewer Expertise:

Computational Biology

I confirm that I have read this submission and believe that I have an appropriate level of expertise to confirm that it is of an acceptable scientific standard.

# Reviewer report: Reviewer response for version 1

**DOI:** 10.21956/wellcomeopenres.29990.r170816

**Published:** 2026-10-07

**Authors:** Mariana Dias Guilardi

**Affiliations:** 1 Interunit Postgraduate Program in Bioinformatics, University of Sao Paulo, São Paulo, São Paulo, Brazil

The authors provide a high-quality chromosome-level genome for *Dermanura phaeotis, *generated from a male sampled in Belize. The assembly includes an assembly at scaffold-level, and 99.4% of the genome was assigned to 30 chromosome pseudomolecules. This work presents an accurate description of the methods, including HiFi and Hi-C, and the results of the assemblies. Results include scaffold assembly and 30 chromosomal pseudomolecules. I suggest minor modifications to improve the text:

- The authors could include in the **Background **the information about cytogenetic data (2n=30).
- **Methods** describe the DNA extraction was performed using liver, however the deposited **NCBI Biosample** (SAMN48779402) includes all tissues (“muscle, liver, heart, testes, spleen, kidney”). It’s important to correct if the sequencing is from a single or multiple tissues either in **Methods **or in the **NCBI Biosample**.
- **Methods **include information about animal’ ethics that should be moved to **Ethics**’ section. The same happens in the description of the animal ID and locality in **Genome sequence report** that should be placed in **Methods**.
- Authors could include what are the benchmarks for high-quality genomes, for example in **Table 1 **or in the legend of **Figure 4**, for comparison to the results obtained in this work.

Are sufficient details of methods and materials provided to allow replication by others?

Yes

Is the rationale for creating the dataset(s) clearly described?

Yes

Are the datasets clearly presented in a useable and accessible format?

Partly

Are the protocols appropriate and is the work technically sound?

Yes

Reviewer Expertise:

Cytogenetics, Bioinformatics, small mammals

I confirm that I have read this submission and believe that I have an appropriate level of expertise to confirm that it is of an acceptable scientific standard.

# Reviewer report: Reviewer response for version 1

**DOI:** 10.21956/wellcomeopenres.29990.r168701

**Published:** 2026-09-30

**Authors:** Guilherme Siniciato Terra Garbino

**Affiliations:** 1 Federal University of Vicosa, Viçosa, State of Minas Gerais, Brazil

This is an important and straightforward paper describing the genome sequence of a Neotropical fruit-eating bat.

- In the Intro, please mention that *Dermanura* is part of subtribe Artibeina, and also would be important to mention that the "small Artibeus" of subgenus/genus *Dermanura* are always recovered as monophyletic. The change to genus level is more subjective than due to paraphyly of Artibeus (sensu lato).
- In the first paragraph please correct "from Sinaloa to Guerroro), " o "from Sinaloa to Guerrero), "
- Please cite de Mammal Diversity Database in the references

Are sufficient details of methods and materials provided to allow replication by others?

Yes

Is the rationale for creating the dataset(s) clearly described?

Yes

Are the datasets clearly presented in a useable and accessible format?

Yes

Are the protocols appropriate and is the work technically sound?

Yes

Reviewer Expertise:

biogeography and systematics

I confirm that I have read this submission and believe that I have an appropriate level of expertise to confirm that it is of an acceptable scientific standard.

# Reviewer report: Reviewer response for version 1

**DOI:** 10.21956/wellcomeopenres.29990.r168699

**Published:** 2026-09-25

**Authors:** Inge Seim

**Affiliations:** 1 Institute of Deep-Sea Science and Engineering, Sanya, China

In their Data Note, de Waal and colleagues report a high-quality genome assembly of a male *Dermanura phaeotis* bat. Overall, I have no major concerns and recommend acceptance of this genome report after the authors address the minor issues outlined below.

 • The sample is described twice, each time with different wording (see **Genome sequence report** section and **Methods**) ... it is a bit confusing

 • Text on animal ethics can be found in **Methods**; it should go into the dedicated **Ethics** section

 • It is surely chromosome confOrmation, not chromosome confIrmation?

 • Chromosome-level instead of chromosomal-level?

 • Some words randomly in bold (e.g., ‘liver’ in **Methods**)

 • **Table 1** lists multiple tissue types (muscle, liver, testes, spleen, kidney), but the Methods section only mentions that liver was used for PacBio HiFi and Hi-C sequencing. The NCBI BioSample entry (https://www.ncbi.nlm.nih.gov/biosample/?term= SAMN48779402) lists all tissues, which is confusing—it seems unlikely they were all pooled before sequencing. Please note that I also pointed out this [potential] issue in another bat manuscript (https://wellcomeopenresearch.org/articles/11-527/v1), but it does not appear to have been addressed yet

 • **Table 2**: Consider using the same number of decimal places throughout (e.g., 43.0 instead of 43 for consistency with other values in Table 2)

 • **Table 2**: Why is 30 in italics, and why is ‘n’ in 2 *n* not?

 • **Figure 3**: remind the reader that it is a male, so that it is clear that 2 *n*=30, not 32

 • **Figures 5 and 6**: Consider inverting the text labels on the *y*-axes for better readability

Are sufficient details of methods and materials provided to allow replication by others?

Yes

Is the rationale for creating the dataset(s) clearly described?

Yes

Are the datasets clearly presented in a useable and accessible format?

Partly

Are the protocols appropriate and is the work technically sound?

Yes

Reviewer Expertise:

comparative genomics

I confirm that I have read this submission and believe that I have an appropriate level of expertise to confirm that it is of an acceptable scientific standard.
